# Spread of *Pseudomonas aeruginosa* ST274 Clone in Different Niches: Resistome, Virulome, and Phylogenetic Relationship

**DOI:** 10.3390/antibiotics12111561

**Published:** 2023-10-24

**Authors:** Gabriela Chichón, María López, María de Toro, Lidia Ruiz-Roldán, Beatriz Rojo-Bezares, Yolanda Sáenz

**Affiliations:** 1 Área de Microbiología Molecular, Centro de Investigación Biomédica de La Rioja (CIBIR), C/Piqueras 98, 26006 Logroño, Spain; 2 Plataforma de Genómica y Bioinformática, Centro de Investigación Biomédica de La Rioja (CIBIR), C/Piqueras 98, 26006 Logroño, Spain; 3 Joint Research Unit “Infection and Public Health” FISABIO-University of Valencia, Institute for Integrative Systems Biology I2SysBio (CSIC-UV), Av. de Catalunya 21, 46020 Valencia, Spain

**Keywords:** ST274, whole genome sequencing, resistome, virulence, motility, pigment, biofilm, *lasR*, cystic fibrosis

## Abstract

*Pseudomonas aeruginosa* ST274 is an international epidemic high-risk clone, mostly associated with hospital settings and appears to colonize cystic fibrosis (CF) patients worldwide. To understand the relevant mechanisms for its success, the biological and genomic characteristics of 11 ST274-*P. aeruginosa* strains from clinical and non-clinical origins were analyzed. The extensively drug-resistant (XDR/DTR), the non-susceptible to at least one agent (modR), and the *lasR*-truncated (by IS*Psp7*) strains showed a chronic infection phenotype characterized by loss of serotype-specific antigenicity and low motility. Furthermore, the XDR/DTR and modR strains presented low pigment production and biofilm formation, which were very high in the *lasR*-truncated strain. Their whole genome sequences were compared with other 14 ST274-*P. aeruginosa* genomes available in the NCBI database, and certain associations have been primarily detected: *bla*_OXA-486_ and *bla*_PDC-24_ genes, serotype O:3, *exoS*^+^/*exoU*^−^ genotype, group V of type IV pili, and pyoverdine locus class II. Other general molecular markers highlight the absence of *vqsM* and *pldA*/*tleS* genes and the presence of the same mutational pattern in genes involving two-component sensor-regulator systems PmrAB and CreBD, exotoxin A, quorum-sensing RhlI, beta-lactamase expression regulator AmpD, PBP1A, or FusA2 elongation factor G. The proportionated ST274-*P. aeruginosa* results could serve as the basis for more specific studies focused on better antibiotic stewardship and new therapeutic developments.

## 1. Introduction

*Pseudomonas aeruginosa* is a ubiquitous Gram-negative microorganism that is adaptable and metabolically versatile and has been found in a wide variety of habitats. This species is a relevant opportunistic pathogen and one of the most frequent causes of acute nosocomial infections [1,2,3]. The increasing prevalence of multidrug-resistant (MDR) *P. aeruginosa* strains makes this bacterium difficult to treat, and it is consequently associated with a high risk of mortality.

*P. aeruginosa* strains have the capacity to develop resistance to antibiotics by the selection of genomic mutations and by exchange of transferable resistance determinants [4]. Additionally, their pathogenicity is associated with the expression of multiple virulence factors that enable evasion of the host response, such as lipases, proteases, rhamnolipids, pyocyanin, pyoverdine, catalases, and exopolysaccharides, as well as biofilm production. Most of them are under the control of quorum-sensing (QS) systems (including Las, Rhl, QscR, Pqs, and Iqs), with LasIR and RhlIR being the most dominant regulatory circuits [5,6].

*P. aeruginosa* commonly infects the lungs of cystic fibrosis (CF) patients, and the infections typically progress from the intermittent acquisition of single environmental strains to an extensive genetic and phenotypic adaptation to the lung environment. The characteristics associated with the transition from “acute” to a “chronic” pulmonary pathogen in CF include the downregulation of some virulence factors (motility and production of pigments, rhamnolipids, and proteases), mucoid morphotype, increased biofilm formation, and upregulation of exopolysaccharide expression, as well as the reduced quorum-sensing pathways [5,7]. Therefore, *P. aeruginosa* is practically impossible to eradicate in the chronic phase of infection [8,9].

Some specific sequence types (ST) of *P. aeruginosa* have been commonly found associated with certain antibiotic resistance, virulence, or infective characteristics [10]. In previous epidemiological CF Spanish studies, the genetic background of obtained *P. aeruginosa* isolates showed high genetic variability, and ST395 and ST274 were identified as endemic clones [2]. Moreover, whole genome sequence analyses have revealed the emergence of CF-adapted epidemic clones, which may result from a limited number of specific mutations with pleiotropic effects [11].

The international epidemic high-risk clone ST274 and its clonal complex (CC274) are mostly associated with hospital settings and appear to colonize CF patients worldwide [10,12,13,14,15,16,17,18,19]. Thus, the majority of studies focus on characterizing globally disseminated clones in clinical settings, but few on non-clinical strains [15,20,21]. The aim of this work was to analyze the biological and genomic characteristics of ST274-*P. aeruginosa* strains from different origins, to set the path in understanding the relevant mechanisms for its success in the CF setting.

## 2. Results

### 2.1. Resistance Phenotype

Nine of 11 ST274-*P. aeruginosa* strains were susceptible to all anti-pseudomonal agents tested (classified as multiS in this work). One non-clinical strain, recovered from a fecal sample of a healthy volunteer, showed intermediate resistance to ceftazidime and imipenem and was classified as modR. The remaining clinical strain showed non-susceptible phenotypes to piperacillin–tazobactam, ceftazidime, cefepime, imipenem, meropenem, doripenem, gentamicin, ciprofloxacin, levofloxacin, and aztreonam, and it was classified as extensively drug-resistant (XDR) and difficult-to-treat resistant (DTR) strain (Table 1 and Supplementary Materials Table S1). All strains were susceptible to colistin, cefiderocol, ceftazidime–avibactam, and ceftolozane–tazobactam. No isolate showed class A carbapenemase, extended-spectrum beta-lactamase (ESBL), or metallo-beta-lactamase (MBL) phenotypes. AmpC hyperproduction was observed in two strains (Table 1 and Supplementary Materials Table S1).

### 2.2. Serotyping and Pulsed-Field Gel Electrophoresis (PFGE)

Table 1 presents the serotypes determined by agglutination, with the O:3 serotype found in all strains except four non-typeable strains.

**Figure 1:**
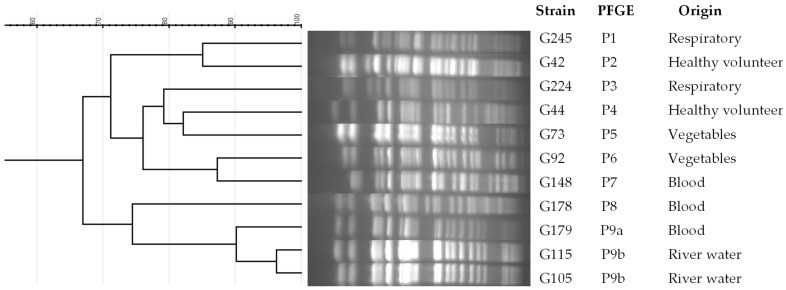
Dendrogram of PFGE patterns in ST274-*P. aeruginosa* strains from different origins. PFGE patterns were analyzed by GelJ v2 program, using the Dice coefficient.

Nine PFGE patterns were observed among the 11 ST274-*P. aeruginosa* strains studied (Figure 1 and Table 1). An indistinguishable pattern was found between two strains from river water samples that were closely related (more than 90% of identity) to the XDR-clinical strain. No association was detected by origin or resistance phenotype.

### 2.3. Detection of Virulence and Quorum-Sensing Genes

The definition of virulotypes was based on the detection of the 14 genes involved in virulence or quorum-sensing (*exoS*, *exoU*, *exlA*, *exoY*, *exoT*, *exoA*, *lasA*, *lasB*, *aprA*, *rhlAB*, *rhlI*, *rhlR*, *lasI*, and *lasR* genes). Two virulotypes were detected (Table 1). All strains harbored *exoS*, *exoY*, *exoT*, *exoA*, *lasA*, *lasB*, *aprA*, *rhlAB*, *rhlI*, *rhlR*, *lasI*, and *lasR* genes, whereas the strain G245 had the *lasR* gene truncated by the IS*Psp7* element, an IS*30*-family insertion sequence. The *exoU* and *exlA* genes were absent in all of the strains.

### 2.4. Biofilm Formation

**Figure 2:**
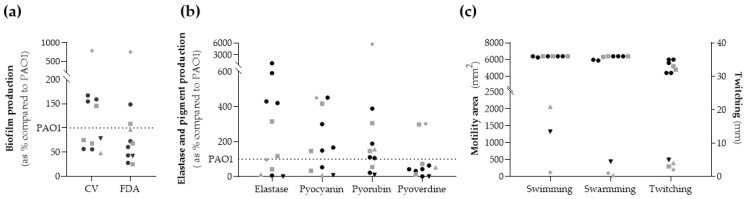
Phenotypic assays of biological parameters. (**a**) Biofilm production: biofilm biomass (CV) and metabolic activity inside the biofilm (FDA). (**b**) Elastase and pigments production. (**c**) Motility activity: swimming and swarming in mm^2^ (left axis) and twitching in mm (right axis). The dotted line represents reference strain *P. aeruginosa* PAO1 value = 100%. Black symbol (dots and triangle) represents non-clinical strains, and grey symbols (squares, triangle and rhombus) represent clinical strains. The grey triangle corresponds to the XDR strain, the black triangle to the modR strain, and the grey rhombus to the *lasR*-truncated strain.

The production of biofilm biomass (crystal violet (CV) method) and the quantification of bacterial metabolic activity (fluorescein diacetate (FDA) method) were measured in comparison to the control strain PAO1, and the obtained results are shown in Figure 2a and Supplementary Table S1. Six strains (55%, three clinical and three non-clinical) showed less biomass production than PAO1, and eight strains (73%) had less bacterial metabolic activity than PAO1. The XDR strain showed low biofilm production (47.92% in CV and 96.41% in FDA), whereas the *lasR*-truncated strain had very high biofilm production (787% in CV and 753% in FDA).

### 2.5. Elastase Activity and Pigment Production

Results obtained for elastase and pigment production are summarized in Figure 2b and Supplementary Table S1. The elastase production was higher than PAO1 in six strains (55%). The non-clinical *P. aeruginosa* strains produced more than 400%, except for both strains from healthy volunteers whose productions were lower than 7%. Additionally, the XDR strain had a low elastase production compared to PAO1 (11%).

Seven (64%) and eight (73%) strains showed higher pyocyanin and pyorubin production than PAO1, respectively. However, only two strains (G178 and G245) had a higher pyoverdine production than PAO1 (18%). The XDR strain exhibited a low production of pyocyanin pigment (8%), and the modR strain showed a very low production for all pigments. On the other hand, the *lasR*-truncated strain showed exceptionally high pyorubin (5714.29%), pyocyanin (450%), and pyoverdine (302%) production.

### 2.6. Motility

High swimming and swarming (area ranges 5892–6400 mm^2^) and twitching motility (diameter ranges 31–35 mm) were found in all ST274-*P. aeruginosa* strains except for three. These three strains were modR, XDR, and *lasR*-truncated ones that showed low motility values (area ≤ 2500 mm^2^ ranged from 34 to 2100 mm^2^, and twitching diameter ≤ 5 mm). G178 strain showed high swimming and swarming values (6400 mm^2^) but low twitching motility (3 mm) (Figure 2c and Supplementary Materials Table S1).

### 2.7. Genome Properties of ST274-P. aeruginosa Strains from Different Origins

The genome properties of the 11 studied ST274-*P. aeruginosa* strains and their assembly parameters are detailed in Table 2. Genome sizes ranged from 6,241,882 to 6,805,979 bp with 66.04–66.54% GC content. The total number of contigs ranged between 46 and 109, and the number of genes oscillated from 5734 to 6267.

#### 2.7.1. Phylogenetic Analysis

Phylogenetic analysis was performed with a total of 27 *P. aeruginosa* strains, comparing our 11 studied ST274-*P. aeruginosa* strains with 14 reference ST274-*P. aeruginosa* genomes downloaded from the NCBI database, and the genomes of control strains *P. aeruginosa* PAO1 and PA14 (Supplementary Materials Table S2). The core genome and the SNP distance depended on the addition of PA14 and PAO1 genomes. Thus, the core genome of these 27 *P. aeruginosa* strains included 4,538 CDS with 90% homology and 90% coverage. By analyzing the SNPs, a range of 1 to 29,065 SNPs was detected among the 27 sequences; within the range, there were 1 to 4204 SNPs among all 25 ST274-*P. aeruginosa* sequences and from 1 to 3665 among the 11 ST274-*P. aeruginosa* sequences in this work (Supplementary Materials Table S3).

**Figure 3:**
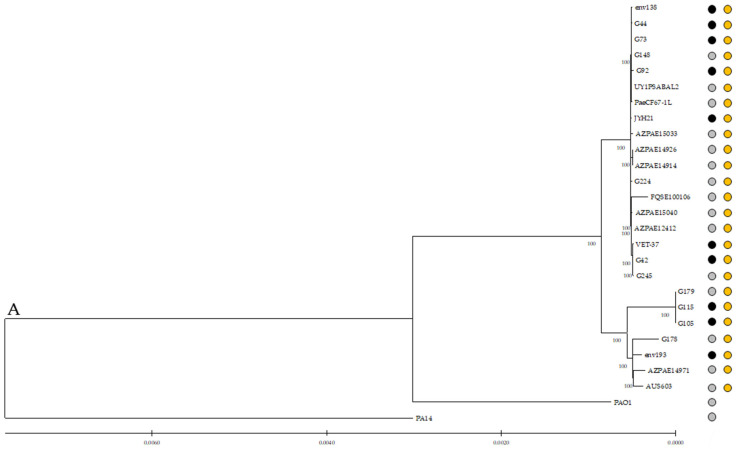
Core genome phylogenetic reconstructions of *P. aeruginosa*. Genetic relationship between all 25 ST274-*P. aeruginosa* analyzed and control strains PAO1 and PA14. The phylogenetic tree was constructed using IQ-Tree 1.6.12. Orange dots represent the ST274 strains, and grey and black dots represent clinical and non-clinical strains, respectively. Bootstrap values, in percent, are shown on the internal nodes.

The core genome alignment was used to generate a phylogenetic tree (Figure 3), which showed no differences among strains from different origins or geographical areas. This representation can determine that ST274 is phylogenetically closer to PAO1 than to PA14 control strain. It is also remarkable to observe that the three PFGE-related strains (G105, G115, and G179) are clustered in the same clade, which is close to G178 and the reference env193 (isolated from the horse water trough, USA), AZPAE14971 (intra-abdominal tract infection, China), and AUS603 (CF, Australia) genomes.

Pangenome was determined using Roary in all 25 ST274-*P. aeruginosa* strains and the control strains PAO1 and PA14. A total of 158,151 genes were analyzed, detecting between 5132 to 5296 core genes and 2594 unique genes. These unique genes represented 1.64% of the total number of genes analyzed and ranged from 0 (G105, G179, and env193) to 533 (FQSE100106) (Supplementary Materials Table S4).

There were 60 ORFs detected in all 25 ST274-*P. aeruginosa* strains, but not in control strains PAO1 and PA14, and were classified into 17 COG categories according to the EggNOGmapper program (Supplementary Materials Table S4).

#### 2.7.2. Resistome

The presence of antimicrobial-acquired resistance genes and the mutational resistome was analyzed in the 11 ST274-*P. aeruginosa* strains from this work and the 14 reference ST274-*P. aeruginosa* genomes.

The *bla*_PDC-24_ and *bla*_OXA-486_ genes (β-lactam resistance) were detected in all isolates, except AZPAE15040 which harbored the *bla*_PDC-69_ variant. The *aph(3′)-IIb* (aminoglycoside resistance), *catB*7 (phenicol resistance), and *fosA* (fosfomycin resistance) genes were detected in all strains, excluding AZPAE14971 strain which lacked the *aph(3′)-IIb* gene. Two reference strains (AZPAE14926 and AZPAE14914) had additionally *aph(3″)-Ib*, *aph(6)-Id*, *sul1*, *sul2*, *bla*_AER-1_, and *floR_1* genes (Supplementary Materials Table S5).

**Figure 4:**
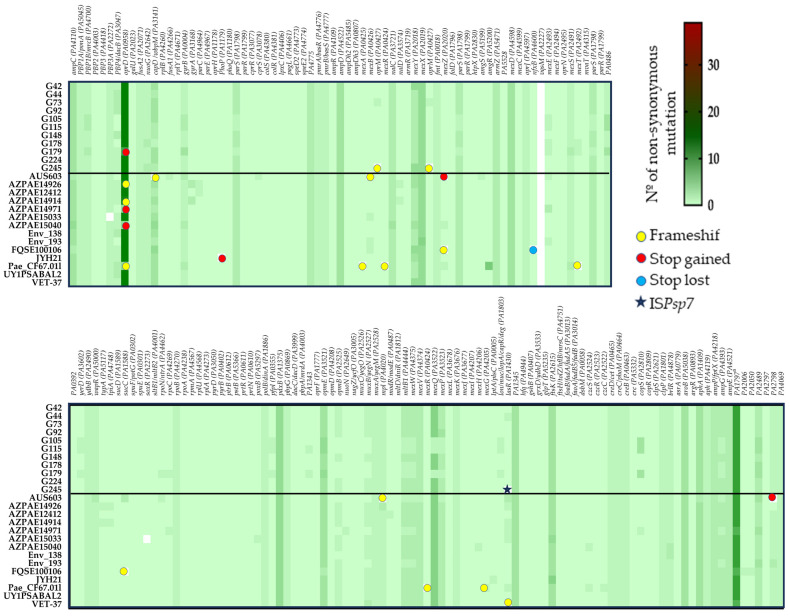
Heatmap generated with GraphPad Prism 8 represents the mutational resistome (non-synonymous mutations in 170 genes involved in antimicrobial resistance) among the 11 studied ST274-*P. aeruginosa* strains and the 14 reference ST274-*P. aeruginosa* genomes. The color scale represents the number of mutational events with respect to the PAO1 genome, except *mexT* which was compared to PA14.

To determine the mutational resistome, the presence of non-synonymous substitutions was analyzed among a dataset of 170 PAO1 genes involved in antimicrobial resistance (Supplementary Materials Table S6). Supplementary Materials Table S7 presents the aminoacidic changes detected in each strain. The mutational resistome pattern was very similar between the ST274 strains (from this work and the reference strains) (Figure 4). The wild-type PAO1 sequence was detected in 69 genes (41%) of all studied ST274-*P. aeruginosa*; however, at least one missense mutation was observed in 57 genes (34%), 13 of which presented three or more non-synonymous substitutions. The highest number of aminoacidic changes was found, independently of the resistance phenotype of the strains, in PA1797, OprD, MexX, PdxB, and MexQ proteins.

The results highlight the absence of *vqsM* gene and the presence of the same change/s in 26 genes (15.3%) of all ST274-*P. aeruginosa* strains, antimicrobial susceptible and resistant ones, that include substitutions in proteins such as the two-component sensor-regulator systems PmrAB (substitutions L71R and Y345H, respectively) and CreBD (E128G and A397V, respectively); the beta-lactamase expression regulator AmpD (R11L, G148A, D183Y); the penicillin-binding protein 1A (A615_D616insP); or the elongation factor G FusA2 (G695A) (Figure 4 and Supplementary Materials Table S7).

The XDR strain (G179) showed different missense mutations in comparison to our remaining 10 multiS and modR ST274-*P. aeruginosa* strains. The differences were detected in 8 of the 167 studied proteins: OprD porin (9 substitutions and a premature stop codon at position W277) implicated in carbapenem resistance; DacB/PBP4 (deletion of 12 amino acids, N454_L465del) involved in beta-lactam resistance; GyrB (S466F) related to quinolone resistance; CapD (T27S; S51G; T507A) involved in O-antigen biosynthesis and previously related with aminoglycoside resistance; MexT (T157S) and MexS (D249N; G275S) multidrug efflux pump MexEF-OprN regulators; RpoC (E386K) a DNA-directed RNA polymerase beta chain; and RpoN (insertion of 4 amino acids, I180insSLEE) the RNA polymerase sigma-54 factor that regulates many virulence genes and is linked to antibiotic resistance (Supplementary Materials Table S7). In addition, the same changes in 16 proteins were only detected in the XDR strain and its two closely related strains (G105 and G115) (Figure 4), with the exception of those substitutions in MexE, MltB1, MexW, AmpG, PA2489, and PA4069 proteins that were also detected in G178 and the reference env193, AZPAE14971, and AUS603 genomes.

The *lasR*-truncated strain (*P. aeruginosa* G245) showed unique amino acid changes in NuoG (T484A, S527N) and OprM (T173fs).

Concerning the mutome panel (Supplementary Materials Table S8), the wild type of *mutS* and *mutL* genes was detected in all 25 ST274 studied. On the other hand, the amino acidic change D61N in MutY protein was detected in 19 strains (76%), the E236D in MutT of 18 strains (72%), and D876E in PolA of all but VET-37 strain (96%).

According to the PlasmidFinder database, none of the strains harbored plasmids.

#### 2.7.3. Detection of Virulence and Quorum-Sensing Genes

The serotype O:3 was detected in all ST274-*P. aeruginosa* strains using in silico serotyping with PAst 1.0.

**Figure 5:**
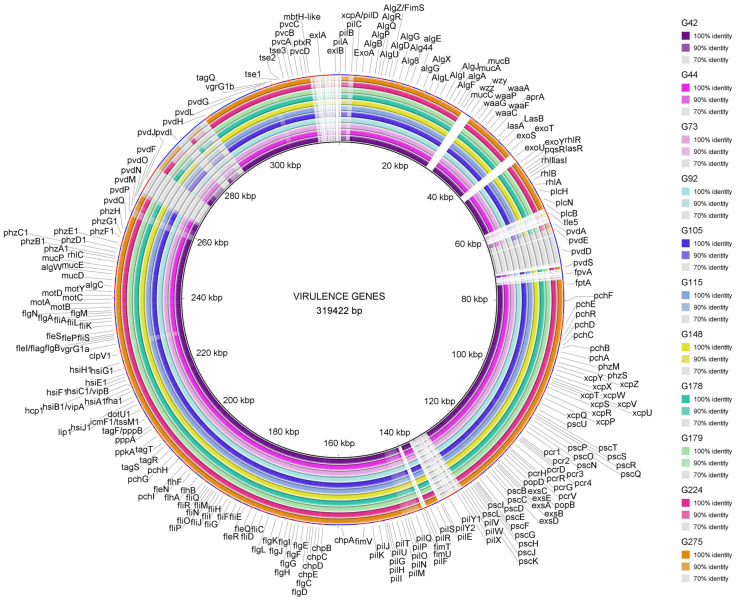
Virulence genes detected among the 11 studied ST274-*P. aeruginosa* strains. The BRIG Tool was used to represent the genes and their identity, using the reference *P. aeruginosa* PAO1 genome (except for *exlA* and *exlB* genes that were compared to PA7 and *exoU* to PA14 genomes). Figure legend includes the name of the studied *P. aeruginosa* strains and the color according to the percentage of identity detected.

The presence and alterations of a dataset of 247 virulence genes of PAO1, *exoU* gene of PA14, and *exlA* and *exlB* genes of PA7 (Supplementary Table S9) were analyzed in the 11 ST274-*P. aeruginosa* strains and the 14 reference ST274-*P. aeruginosa* genomes. Figure 5 shows the comparison of the virulence genes between PAO1 and our 11 ST274-*P. aeruginosa* strains. Supplementary Table S10 summarizes the complete results obtained with the 25 ST274-*P. aeruginosa* genomes.

No changes were observed in 91 out of the 250 (36.4%) genes analyzed. On the other hand, all 25 ST274 strains shared the following characteristics in comparison with PAO1 genes: 16 genes were absent (*pilA*, *pilE*, *pilV*, *pilW*, *pilX*, *pilY1*, *pilY2*, *fimT*, *fimU*, *pldA*/*tleS*, *fpvA*, *wzz*, *wzy*, *exlA*, *exlB*, and *exoU*), and 13 genes, mostly involved in pyoverdine and type IV pili biosynthesis (*pvdL*, *pvdE*, *pvdD*, *pvdJ*, *pvdI*, *pvdF*, *pvdM*, *pvdP*, *pvdA*, *pchI*, *pilP*, *pilN*, and *pilC*), showed more than 10 equal missense changes. These genes were studied in depth as explained below.

The same mutational patterns were detected in 68 proteins (27%) of all ST274 strains, independently of their clinical/non-clinical or geographical origins. On the other hand, PscP (translocation protein in type III secretion) and LasR (quorum-sensing regulator) showed the highest number of different mutational patterns (10 and 9, respectively) among the 25 ST274-*P. aeruginosa.* Additionally, premature stop codons, deletions, or insertions were detected in six genes (*pilC*, *pchF*, *pvdM*, *pcrH*, *pscK*, and *toxA*) in all strains. This highlights the results observed in ToxA, the exotoxin A precursor, where two mutational patterns including deletions were found: (i) T4I, F22S, G386_A388del, I432V, D500A, detected in ST274-*P. aeruginosa* G105, G115, G179, and G178 and the reference env193, AZPAE14971 and AUS603 strains, and (ii) T4I, F22S, A58_T62del, and I432V observed in the remaining 18 strains.

The XDR strain (*P. aeruginosa* G179) showed unique amino acid changes in PilN (V30L, A31V, A33G, D40G, F43L, T44N, A45T, N49H, N51G, K75R, and a premature stop codon at position Q80) and in RhlR (Y72C) and shared the same missense mutational patterns in 23 genes only with *P. aeruginosa* G105, G115, G178, env193, AZPAE14971, and AUS603 strains.

The *lasR*-truncated strain (*P. aeruginosa* G245) had unique mutational patterns in FleQ, PchH, PchE (D6fs), and PvdS proteins. The ModR strain (*P. aeruginosa* G44) only showed the amino acid change C79I in LasR in comparison with the remaining ST274-*P. aeruginosa* strains.

All 40 Type III Secretion System (T3SS)-related genes and their translocated effectors were detected in all ST274 strains, with the exception of *exoU* gene. The genes involved in flagella, phenazines, and alginate were mostly highly conserved and homologous among all ST274 strains; however, type IV pili (T4P), pyochelin, and pyoverdine biosynthesis genes were more variable. Analyzing the 23 T4P biosynthesis genes (Supplementary Materials Table S9), there were 9 absent genes (*pilA* and the *fimU*/*T*/*pilV*/*W*/*X*/*Y1*/*Y2*/*E* gene cluster), and at least one missense mutation was observed in 11 genes of all ST274 strains, showing a high polymorphism in proteins, such as PilC, PilN, PilO, or PilP (from 8 to 50 modifications) (Supplementary Materials Table S10). The major subunit of T4P is a protein encoded by the *pilA* gene. This gene has five pili alleles that are found at a conserved chromosomal locus between the adjacent *pilB* and *tRNA*-Thr genes [23]. By analyzing the *pilB*/*tRNA*-Thr region in all ST274 strains against GenBank sequences using BLAST, we found that *pilA* belongs to T4P group V (*pilA_V_*), as well as the *fimU*/*T*/*pilV*/*W*/*X*/*Y1*/*Y2*/*E* gene cluster. For that reason, it was not possible to detect it when compared to PAO1-*pilA* which belongs to T4P group II (*pilA_II_*) (parameters used included >90% similarity).

**Figure 6:**
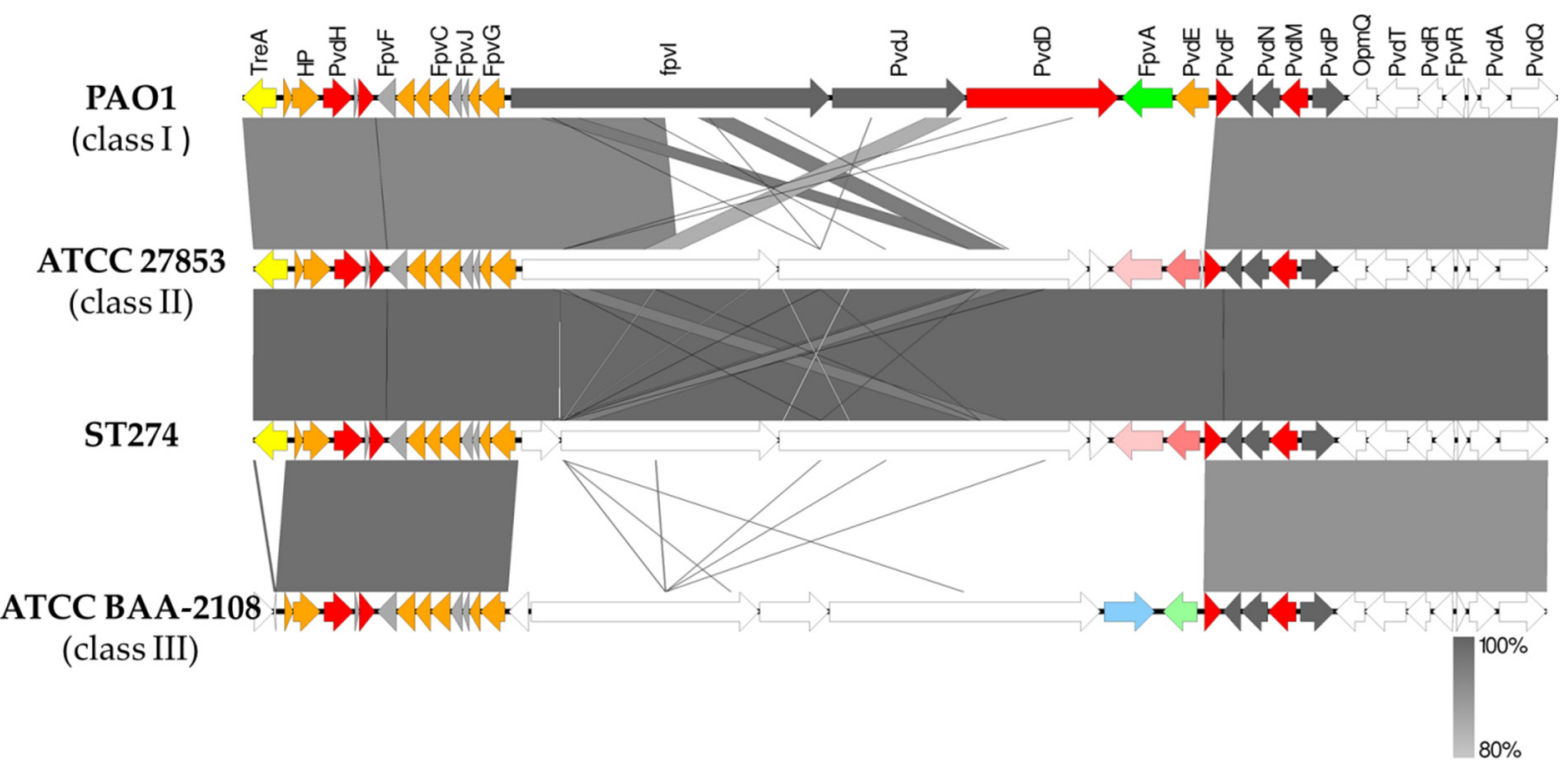
Schematic representation of the genomic differences found in the pyoverdine region between *P. aeruginosa* PAO1 (class I), ATCC27853 (class II), ATCC BAA-2108 (class III), and ST274 *P. aeruginosa* strains. Colors represent the localization of genes according to the *Pseudomonas* webpage (www.pseudomonas.com (accessed on April 2023)): orange, cytoplasmic membrane proteins; red, cytoplasmic proteins; green, outer membrane proteins; grey, proteins of unknown function. Furthermore, the yellow arrow indicates *treA* gene (absent in class III); light pink represents another iron receptor different to FpvA, and dark pink represents a cyclic peptide export ABC transporter; blue, light green arrows indicate two different proteins located in that region in class III pyoverdine region and white arrows represent hypothetical proteins (unknown function). Easyfig v2.2.5 and BLAST v2.10.0 were used to build the figure.

Pyoverdine biosynthesis in *P. aeruginosa* is a complex process involving at least 16 different proteins (Supplementary Materials Table S9). In all ST274 strains, *pvdA*, *pvdP*, *pvdD*, *pvdE*, *pvdJ,* and *pvdI* were highly divergent genes compared with PAO1 ones, and *fpvA* gene (ferripyoverdine receptor) was absent. When the region between *treA* and *pvdQ* was compared to GenBank genomes using BLAST, an identity higher than 80% was detected between our ST274 strains and multiple sequences, including the reference PAO1 (class I), ATCC27853 (class II), and the ATCC BAA-2108 (class III) strains. Figure 6 shows the alignment of the pyoverdine region of an ST274 strain in comparison with class I, class II, and class III pyoverdine-producing strains. All ST274-*P. aeruginosa* presented a pyoverdine locus class II.

## 3. Discussion

Different studies have revealed a high genetic diversity among *P. aeruginosa* isolates infecting CF patients. However, ST274, defined as an endemic clone in Spain and a CF epidemic clone worldwide, has been detected infecting multiple CF patients from Europe, America, Asia, and Australia [2,10,12,13,14,15,16,18,19]. Most of the reported studies characterized the ST274-*P. aeruginosa* isolated from CF patients or associated with hospital settings worldwide, although the majority of chronic CF lung infections are thought to be the result of colonization by *P. aeruginosa* from environmental sources. The investigation on the ST274-*P. aeruginosa* behavior, including origin, antibiotic resistance, virulence, pathogenicity, molecular typing, or environmental/infection adaptability, is necessary to know the molecular markers and biological characteristics that could explain the clonal success of the ST274 clone. In addition, the assessment of the genetic and biological markers underlying clonal success must be performed in a range of not only both resistant and susceptible strains but also both clinical and non-clinical strains.

In the current study, five clinical and six non-clinical ST274-*P. aeruginosa* strains have been deeply analyzed, and their whole genome sequences were compared with others included in databases. Regarding antimicrobial resistance, 9 out of 11 ST274-*P. aeruginosa* strains were multiS, one strain was modR, and the remaining strain was XDR and DTR. High-risk clones ST235, ST175, and ST111 are usually associated with MDR/XDR clinical strains; however, as our results show, in agreement with previous studies reported [21,24], these resistance phenotypes are not so frequent among non-clinical or ST274 *P. aeruginosa* strains. The unique XDR and DTR strain (G179) in our work was isolated from a blood sample, showed hyperproduction of AmpC, and possessed a different mutational resistome in comparison to the remaining 10 ST274-*P. aeruginosa* strains. G179 resistome showed missense mutations affecting porins and membrane proteins, multidrug efflux pumps, O-antigen biosynthesis, quinolone targets, and regulators (i.e., OprD, DacB/PBP4, GyrB, CapD, MexT, MexS, RpoC, and RpoN proteins), which likely explain the observed resistance pattern. The mutational inactivation of *oprD* is the most frequent cause of carbapenem resistance in *P. aeruginosa*, and the inactivation of *dacB,* which correlated with the AmpC overexpression, causes the β-lactam resistance [25,26,27]. RpoN regulates nitrogen assimilation, quorum sensing, motility, biofilm formation, and other virulence factors, but its regulation was also linked to *P. aeruginosa* tolerance to several antibiotics [28,29]. The insertion of four amino acids in the RpoN of the G179 strain probably induced a partial or total loss of RpoN function which is a relatively common mechanism of pathoadaptation to the CF lungs [30,31]. This strain was also classified as DTR, referring to resistance to all first-line antibiotics that include β-lactams and fluoroquinolones, and associated with higher mortality [32,33]. According to international guidelines [33], ceftolozane–tazobactam, ceftazidime–avibactam, or imipenem–relebactam are recommended for treating DTR *P. aeruginosa,* and our DTR ST274-*P. aeruginosa* strain was susceptible to all of them.

During an infection, *P. aeruginosa* has to adapt to a new scenario that includes the host immune response, antibiotics, and a different environment and substrate composition. There are multiple references analyzing the *P. aeruginosa* adaptive processes, and it is commonly observed that the gradual downregulation or loss of the function of many genes occurs, usually associated with bacterial quorum-sensing and pathogenicity [5,7,31,34,35]. Persistent colonization and chronic infection are eventually associated with resistance development, loss of O-antigen, biofilm production, and loss of motility and virulence factors. Among our analyzed 11 ST274-*P. aeruginosa* strains, the XDR, modR, and *lasR*-truncated strains showed a chronic infection phenotype characterized by loss of serotype-specific antigenicity and low motility. The remaining eight ST274-*P. aeruginosa* strains were fully motile in agreement with a general wild-type phenotype. Furthermore, the XDR and modR strains presented low production of pigments and reduced biofilm formation and values that were very high in the *lasR*-truncated strain. The *lasR* gene is involucrate in the control of quorum-sensing systems and is generally considered at the top of the regulatory hierarchy [36,37]. LasR showed the C79Y change in the modR strain recovered from a fecal sample of a healthy volunteer; whereas in the *lasR*-truncated strain, isolated from a bronchial aspirate of a patient with chronic bronchitis, the *lasR* was truncated by the IS*Psp7* element. *P. aeruginosa* is frequently observed to gain mutations in *lasR* gene during chronic infections, including chronic CF lung infections [31,38,39], but a high prevalence of *lasR*-defective *P. aeruginosa* has been detected from not only clinical samples but also non-clinical origins [40,41,42]. This fact could suggest that the loss of *lasR* function gives a selective pathogenic advantage and minimizes fitness cost at a bacterial population level even outside the clinical contexts. Furthermore, there are multiple evolution studies analyzing the in vitro and in vivo effect of *lasR* mutants, and they have been detected that even the nutritional environment is sufficient to select those mutants, and not all of them involve a LasR-defective phenotype [39,41,43,44]. Thus, *lasR* mutants are neither atypical nor restricted to clinical isolates, and additionally, the *lasR*-loss could be offset by other interactive quorum-sensing systems and/or global regulators, such as MvaT, RpoN, and RpoS.

The *vqsM* and *pldA*/*tleS* genes were not detected among the 25 studied ST274 sequences. VqsM is an AraC family transcriptional regulator which activates the *las* quorum-sensing system and T3SS gene expression [45]. *pldA*/*tleS* gene codifies a phospholipase D1 involved in bacterial pathogenesis and persistence [46]. However, it has been reported that not all strains of *P. aeruginosa* carry *vqsM* or *pldA* genes; therefore, their relative infective contributions appear to be strain-specific [45,46].

According to core genome analysis, the phylogenetic tree showed all 25 ST274-*P. aeruginosa* interspersed and grouped in two phylogenetic clades independently of their antimicrobial resistance or origin, supporting the idea of the absence of a geographical or origin barrier for lineage evolution. *P. aeruginosa* G105, G115, G178, G179, env193, AZPAE14971, and AUS603 strains, which were recovered from clinical and non-clinical samples, shared the wild-type sequences of *mutS*, *mutL*, and *mutT* mutator genes, and the same missense mutational patterns in 7 genes of the resistome (i.e., *fosA*, *mexE*, *muxB*, *mltB1*, *mexW*, *ampG*, and *PA2489*) and 23 genes of the virulome (e.g., *toxA*, *rhlC*, *lasI*, or *popN*).

Interclonal sequence variation is low in the *P. aeruginosa* core genome [47]. Indeed, the genome clusters with the highest level of sequence diversity in *P. aeruginosa* are the pyoverdine locus, the flagellar regulon, T4P *pilA*, the T3SS effector proteins, and the O-antigen biosynthesis locus. Each cluster is present in all strains, but their involved genes are highly divergent between strains. This phenomenon could be a result of “diversifying selection”, a type of selection that maintains multiple alleles in the population [47]. In this line, and according to the results obtained in our work, the genes involved in flagella were mostly highly conserved and homologous among all ST274 strains; however, T4P and siderophores (pyoverdine and pyochelin) biosynthesis genes were highly divergent compared with PAO1 ones. T4P is associated with a number of biological activities in bacteria, such as twitching motility, bacteriophage sensitivity, attachment to biotic and abiotic surfaces, biofilm development, and DNA uptake [23]. In *P. aeruginosa*, T4P is composed of a single-type IVa pilin (T4aP) protein encoded by the *pilA* gene, for which five distinct pilin alleles (Groups I–V) have been previously reported in *P. aeruginosa* [23,48]. PAO1 strain possesses group II pilin, PA14 harbors group III, and all ST274 strains of our study presented a group V. Group III and V pilins have an unusual architecture and diverged from a common ancestor [23,49]. The “minor” pilins are a set of low-abundance pilin-like proteins encoded by the *fimU*/*T*/*pilV*/*W*/*X*/*Y1*/*Y2*/*E* gene cluster. In all ST274 strains, they were not detected compared with the PAO1 sequence, as observed in other works [50]. However, it has been also described that both groups III and V have identical minor pilin genes [51], which have less than 75% similarity with the group II operon [52]. In addition, the tandem tRNA genes located between the major and minor pilin operons appear to provide hot spots for genetic insertion. This information together with the fact that some *P. aeruginosa* pilins are more closely related to pilins of distinct bacterial species (e.g., *Dichelobacter nodosus*, *Eikenella corrodens*, *Ralstonia solanacearum*, and *Xanthomonas campestris*) strongly suggest that pilin gene diversity was generated through horizontal genetic transfer [53,54]. Group I and II pilins are the most frequently detected in *P. aeruginosa* strains [23,52,55], and among the scarce descriptions of isolates harboring a group V pilin, no specific differences were reported [23,55]. Further studies could determine the possible relationship between these groups of pilins, motility, and strain-specific characteristics.

In general, every *P. aeruginosa* isolate is able to produce one of three types of pyoverdine [56,57], and all 25 analyzed ST274-*P. aeruginosa* presented a pyoverdine class II. The TonB-dependent ferripyoverdine receptor encoding gene, *fpvA_II_*, is chromosomally located among the most divergent genes in the region and is not a recombination result between types. Outer membrane protein genes such as the pyoverdine receptor *fpvA* are common targets for entry by phage or pyocins. Pyocins are bacteriocins produced by *P. aeruginosa* that kill strains of the same species. This is the case for pyocin S3 which uses FpvAII as a receptor, while the pore-forming pyocin S5 utilizes the FptA ferripyochelin receptor to gain entry to the cells and kill them [58,59]. Pyocins could indeed play a particularly important role during co-colonization of the CF lung by *P. aeruginosa* isolates that possess different pyocin/immunity genes and favor the outcompetition of a particular *P. aeruginosa* clone. Thus, siderophore diversity may be also a resistance mechanism.

In conclusion, we gained insights into the ST274-*P. aeruginosa* isolates population structure using a combination of phenotyping and genotyping techniques, and certain associations have been primarily detected, such as the presence of *bla*_OXA-486_ and *bla*_PDC-24_ genes, O-antigen serotype O:3, *exoS*^+^/*exoU*^−^ T3SS genotype, Group V of T4P, and pyoverdine locus class II. Other general molecular markers or features have also been observed linked to the analyzed ST274-*P. aeruginosa* sequences, highlighting the absence of *vqsM* and *pldA*/*tleS* genes and the presence of the same mutational pattern in genes involved in, for example, two-component sensor-regulator systems PmrAB (substitutions L71R and Y345H, respectively) and CreBD (E128G and A397V, respectively); the exotoxin A (A58 or G386 deletions in ToxA); the quorum-sensing RhlI (S62G, D83E); the beta-lactamase expression regulator AmpD (R11L, G148A, D183Y); the penicillin-binding protein 1A (A615_D616insP); or the elongation factor G FusA2 (G695A).

Whole genome sequence analyses provide detailed genome patterns that might be essential to characterize ST274-*P. aeruginosa* strains, although it is difficult and challenging to correlate genotypic with phenotypic variations. Sequencing alone cannot always predict phenotypic differences, due to the multifactorial nature of *P. aeruginosa*. The reduced number of strains and the intra-clonal variability of the parameters analyzed must be considered among the limitations of this study. This variability could be related to the activation of specific regulatory pathways, horizontal gain or loss of genetic material, or undetected punctual mutations that affect the final expression levels of key genes.

Further work is needed to improve our understanding of the basis for the global success of this and other high-risk clones. A more in-depth understanding of *Pseudomonas* virulence regulation, as well as the use of *P. aeruginosa* transcriptomics, would identify hundreds of genes specifically expressed in vitro or during adaptation to different environments. However, this study could serve as the basis for more specific studies that will prove helpful in designing novel antimicrobial approaches, better antibiotic stewardship, and new therapeutic developments.

## 4. Materials and Methods

### 4.1. Bacterial Strains

Eleven *P. aeruginosa* strains belonging to clone ST274 were selected from the *Pseudomonas* collection of the Molecular Microbiology Area (Centre for Biomedical Research in La Rioja, CIBIR, Logroño, Spain). These strains were obtained from clinical samples of non-CF patients (number of strains), respiratory (2) and blood (3), and from non-clinical samples, fecal samples of healthy volunteers (2), vegetables (2), and river water (2) (Table 1 and Supplementary Materials Table S1). *P. aeruginosa* PAO1, PA7, and PA14 were included as control strains in different assays.

### 4.2. Antimicrobial Susceptibility Testing

Susceptibility testing to 15 antipseudomonal agents was performed by MicroScan WalkAway^®^ microdilution system (MicroScan; Beckman Coulter, Inc., Brea, CA, USA) according to CLSI [22], except cefiderocol, ceftazidime–avibactam, and ceftolozane–tazobactam that were determined by disk diffusion method [22]. Colistin resistance was also screened by colistin broth disk elution [22]. The antimicrobial categories and antipseudomonal agents tested were the following ones: aminoglycosides (gentamicin, tobramycin,), carbapenems (imipenem, meropenem, doripenem), cephalosporins (ceftazidime, cefepime, cefiderocol), fluoroquinolones (ciprofloxacin, levofloxacin), penicillin–β-lactamase inhibitor combinations (piperacillin–tazobactam, ceftazidime–avibactam, ceftolozane–tazobactam), monobactams (aztreonam), and polymyxins (colistin).

Strains were categorized as multiS, modR, MDR (multidrug-resistant), and XDR according to previously published classifications [60,61]. The strains that are non-susceptible to all of the following antibiotics, piperacillin–tazobactam, ceftazidime, cefepime, aztreonam, meropenem, imipenem, ciprofloxacin, and levofloxacin, were considered difficult-to-treat resistant (DTR) [32].

ESBL, MBL, and class A carbapenemase phenotypes were determined by double-disc synergy tests [26]. AmpC hyperproduction was determined by the phenotypic method [62].

### 4.3. Serotyping

Serotype identification was performed by agglutination using commercially monovalent antisera specific for 16 different *P. aeruginosa* O-serotypes (Bio-Rad, Temse, Belgium).

### 4.4. Detection of Virulence and Quorum-Sensing Genes

The presence of *exoS*, *exoU*, *exoY*, *exoT*, *exoA*, *exlA*, *lasA*, *lasB*, *aprA*, *rhlAB*, *rhlI*, *rhlR*, *lasI*, and *lasR* genes was studied by PCR as described previously [20].

### 4.5. Clonal Relationship

PFGE was carried out using the *SpeI* enzyme to digest genomic DNA [63]. PFGE patterns were analyzed by the Java program GelJ v2 using the Dice coefficient [64].

### 4.6. Biofilm Formation

Biofilm assays were performed by crystal violet (CV) staining to quantify the total biofilm biomass and by fluorescein diacetate (FDA) assay to determine the bacterial metabolic activity inside the biofilm structure (viable cells within the biofilm). Both methods were performed in 96-well microtiter plates using an initial inoculum of 10^6^ cfu/mL and measured after 24 h of incubation, as previously published [20]. Absorbance measures were performed using a POLARstar Omega microplate reader (BMG Labtech, Ortenberg, Germany). All experiments were performed in triplicate, including *P. aeruginosa* PAO1 as the control strain. GraphPad Prism (v.8.4.3) was used to build the graphics.

### 4.7. Elastase and Pigment Production

Bacterial strains were grown at 37 °C in Luria-Bertani (LB) broth overnight with shaking. After centrifugation, elastase activity was determined by the Elastin-Congo-Red assay [7], and the chloroform extract method was used to quantify pyocyanin, pyoverdine, and pyorubin pigments [7]. Absorbance/fluorescence measures were performed using a POLARstar Omega microplate reader (BMG Labtech, Ortenberg, Germany). All experiments were performed in triplicate, including *P. aeruginosa* PAO1 as the control strain. GraphPad Prism (v.8.4.3) was used to build the graphics.

### 4.8. Motility

Swarming and swimming motilities were determined by placing 4 µL of bacterial suspension (1 × 10^9^ cells in LB broth) on the middle of 0.5% (swarming) and 0.3% (swimming) LB agar plates (entire plate area was 6400 mm^2^) [20]. Plates were photographed with Chemi Doc system (Bio-Rad, Hercules, CA, USA), after incubation at 37 °C overnight. The images were processed with MotilityJ software (v1.0) [65].

A twitching motility assay was performed starting with overnight-grown bacteria on LB agar plates. Bacteria were stab inoculated with a sterile needle loop to the bottom of the plastic agar interface of LB broth solidified with 1% agar plates. After incubation at 37 °C for 48 h, the agar medium was removed. The twitching diameter was measured after staining with 0.05% (wt/vol) Coomassie brilliant blue (40% methanol, 10% acetic acid) for 50 min. Bacteria were classified as non-motile (diameter ≤ 5 mm), motile (diameter from >5 mm to ≤30 mm), or highly motile (diameter > 30 mm) [66,67].

All assays were performed in triplicate. GraphPad Prism (v.8.4.3) was used to build the graphics.

### 4.9. Whole Genome Sequencing (WGS)

Genomic DNA from the 11 ST274-*P. aeruginosa* strains of this study was extracted using Wizard ^®^ Genomic DNA Purification Kit (Promega, Madison, WI, USA). Quantity and quality were assessed using a Qubit fluorimeter (Thermo Fisher Scientific, Waltham, MA, USA). Libraries were prepared using the TruSeq DNA PCR Free protocol (Illumina, San Diego, CA, USA). Then, the final quality of the libraries was assessed with a Fragment Analyzer (Std. Sens. NGS Fragment Analysis kit 1–6000 bp, AATI) and was quantified by qPCR at the Genomics and Bioinformatics Core Facility (CIBIR). Subsequent sequencing was carried out in an Illumina HiSeq 1500 (Illumina, San Diego, CA, USA).

FastQC v0.11.5 (https://www.bioinformatics.babraham.ac.uk/projects/fastqc/) was used to analyze the quality of raw reads, which were subsequently trimmed and filtered by using Trim Galore v0.4.5 (https://www.bioinformatics.babraham.ac.uk/projects/trim_galore/). Genomes were reconstructed by using PLACNETw [68]. Identification of open reading frames (ORFs) and genome annotation of the assembled genetic elements was performed by using PROKKA v1.13 [69].

Additionally, the genomes of 14 ST274-*P. aeruginosa* strains from different origins were downloaded from the NCBI database (January 2020) to be used as reference genomes in the different comparative analyses (Supplementary Materials Table S2). The core genome was determined as the collection of genes present in the 11 ST274-*P. aeruginosa* strains of this study but also in the 14 reference ST274-*P. aeruginosa* genomes and the control strains *P. aeruginosa* PAO1 and PA14. The parameters used included >90% similarity and >90% coverage, as defined by Lanza et al. (2014) [70]. A phylogenetic tree was constructed with this data using IQ-Tree 1.6.12 [71] and iTol V5.6.3 [72]. Roary v3.11.2 was used to determine and analyze the pangenome, identifying common and unique genes [73], and eggNOG-mapper v2 was used to determine the cluster of orthologous groups (COGs) and functional annotations [74].

The presence of acquired antibiotic resistance genes was evaluated using the following tools: ResFinder V3.2 [75], Comprehensive Antibiotic Resistance Database (CARD) [76], and Antibiotic Resistance Gene-ANNOTation (ARG-ANNOT) [77]. The mutational resistome was determined by analyzing the presence of mutations among a dataset of 170 genes involved in antimicrobial resistance downloaded from the Pseudomonas Genome Database (www.pseudomonas.com (Supplementary Materials Table S6)). For this purpose, variant calling was performed using PAO1 as a reference genome (Snippy V4.5.0) and confirmed with BLAST and CLUSTAL. The heatmap was constructed using the GraphPad Prism (v.8.4.3) program.

Additionally, the mutator phenotype was studied from whole genome sequence data through the analysis of genes from mutome panel [2].

Virulence genes were analyzed using the Virulence Factor DataBase (VFDB) [78], studying a dataset of 247 virulence genes of PAO1, plus *exoU* of PA14, and *exlA* and *exlB* of PA7. These genes are associated with flagella and pili biosynthesis/regulation, protease production, type II, IV, and VI secretion systems, protease IV, enzyme, quorum sensing, alginate production/regulation, and toxins were curated from the VFDB (Supplementary Materials Table S10). BLAST searches were used to match the virulence genes with the bacterial genomes, and BLAST Ring Image Generator (BRIG) [79] was used to represent the percentage identity between these genes and those absent. Moreover, we performed BLAST, Clustal Omega, and variant calling (Snippy V4.5.0) using PAO1 as a reference genome to find the aminoacidic changes in virulence proteins.

PlasmidFinder 1.3 was used to search the plasmid replicon types, and the *Pseudomonas aeruginosa* serotyper (PAst 1.0) was used for in silico serotyping (https://cge.cbs.dtu.dk/services/PAst/).

## Figures and Tables

**Table 1:** Characteristics of the 11 *P. aeruginosa* ST274 from different origins selected for this study.

Strain	Sample	Origin ^a^	PFGE Pattern	Serotype ^b^	Resistance Phenotype ^c^	MIC (mg/L) ^c^												Virulotype ^d^
						PTZ	CAZ	FEP	IMP	MER	DOR	GEN	TOB	CIP	LEV	CT	ATM	
G42	Healthy volunteer	NC	P2	O:3	multiS	≤8	4	4	2	≤1	≤1	4	≤2	≤0.5	≤1	≤2	8	V1
G44	Healthy volunteer	NC	P4	PoliA	modR	≤8	16	4	4	≤1	≤1	4	≤2	≤0.5	≤1	≤2	8	V1
G73	Vegetable (lettuce)	NC	P5	O:3	multiS	≤8	2	4	≤1	≤1	≤1	4	≤2	≤0.5	≤1	≤2	4	V1
G92	Vegetable (chard)	NC	P6	O:3	multiS	≤8	2	4	≤1	≤1	≤1	4	≤2	≤0.5	≤1	≤2	8	V1
G105	River water	NC	P9b	O:3	multiS	≤8	2	4	≤1	≤1	≤1	4	≤2	≤0.5	≤1	≤2	8	V1
G115	River water	NC	P9b	O:3	multiS	≤8	2	4	≤1	≤1	≤1	4	≤2	≤0.5	≤1	≤2	8	V1
G148	Blood	C	P7	O:3	multiS	≤8	2	4	≤1	≤1	≤1	4	≤2	≤0.5	≤1	≤2	4	V1
G178	Blood	C	P8	AutoA	multiS	≤8	2	4	≤1	≤1	≤1	4	≤2	≤0.5	≤1	≤2	4	V1
G179 ^e^	Blood	C	P9a	AutoA	XDR, DTR	>64	>16	>16	>8	8	>4	8	≤2	2	4	≤2	>16	V1
G224	Respiratory	C	P3	O:3	multiS	16	2	2	≤1	≤1	≤1	4	≤2	≤0.5	≤1	≤2	8	V1
G245 ^e^	Respiratory	C	P1	PoliA	multiS	≤8	≤1	≤1	≤1	≤1	≤1	≤2	≤2	≤0.5	≤1	≤2	≤1	V2

^a^ NC, non-clinical; C, clinical origin. ^b^ PoliA, polyagglutination; AutoA, autoagglutination. ^c^ multiS, susceptible to all tested antipseudomonal agents; modR, non-susceptible to at least one agent in 1 or 2 categories; XDR, non-susceptible to all but 1 or 2 categories; DTR, difficult-to-treat resistance. MIC, minimal inhibitory concentration; PTZ, piperacillin–tazobactam; CAZ, ceftazidime; FEP, cefepime; IMP, imipenem; MER, meropenem; DOR, doripenem; GEN, gentamicin; TOB, tobramycin; CIP, ciprofloxacin; LEV, levofloxacin; CT, colistin; ATM, aztreonam. Resistance values according to CLSI breakpoints (2022) [22] are marked with dark grey shadows, and the intermediate resistance values in light grey shadows. ^d^ Definition of virulotypes was based on the detection of *exoS*, *exoU*, *exlA*, *exoY*, *exoT*, *exoA*, *lasA*, *lasB*, *aprA*, *rhlAB*, *rhlI*, *rhlR*, *lasI*, and *lasR* genes. Virulotype V1: the strains amplified *exoS*, *exoY*, *exoT*, *exoA*, *lasA*, *lasB*, *aprA*, *rhlAB*, *rhlI*, *rhlR*, *lasI*, and *lasR* genes. Virulotype V2: this strain showed the virulotype V1 but with *lasR* truncated by IS*Psp7* (*lasR*ΔIS*Psp7*). ^e^ AmpC hyperproducer strains.

**Table 2:** General features of the genomes of the 11 studied ST274-*P. aeruginosa* strains.

Strains	Reads	Assembly Parameters				Genetic Elements						
		Number of Contigs	Contig Maximum Length (bp)	Total Bases in Contigs > 1 kb	N50 (bp)	Genome Size (bp)	GC Content (%)	CDS	Genes	rRNA	tRNA	tmRNA
G42	5,890,730	87	726,697	6,748,134	353,893	6,757,744	66.04	6166	6241	3	71	1
G44	5,143,091	76	739,379	6,550,288	612,825	6,556,612	66.29	6000	6076	3	72	1
G73	4,765,846	66	1,027,638	6,349,927	704,215	6,355,219	66.47	5786	5861	3	71	1
G92	6,001,411	68	1,029,741	6,402,390	726,878	6,410,342	66.42	5840	5913	3	69	1
G105	5,647,144	46	768,785	6,241,289	621,817	6,241,882	66.54	5659	5734	3	71	1
G115	5,060,730	62	768,777	6,302,900	621,730	6,309,215	66.43	5741	5816	3	71	1
G148	5,581,661	69	931,567	6,423,973	695,550	6,430,217	66.35	5856	5934	6	71	1
G178	17,116,583	59	1,439,870	6,404,614	606,103	6,412,019	66.43	5825	5901	4	71	1
G179	7,309,267	78	729,747	6,244,365	427,443	6,251,772	66.53	5662	5739	3	73	1
G224	5,613,901	61	1,454,566	6,350,601	804,729	6,356,169	66.48	5821	5896	3	71	1
G245	11,589,531	109	726,712	6,794,368	409,397	6,805,979	66.07	6192	6267	3	71	1

## Data Availability

All datasets are available. The whole genome data for the 11 ST274-*P. aeruginosa* strains have been deposited at NCBI using BioProject number PRJNA678143. The raw sequencing data were deposited at NCBI’s Sequence Read Archive (SRA) (SRR13053896 to SRR13053906).

## Supplementary Materials

The following supporting information can be downloaded at https://www.mdpi.com/article/10.3390/antibiotics12111561/s1, Table S1. Characteristics of the 11 studied ST274-*Pseudomonas aeruginosa* recovered from different origins; Table S2. Characteristics of the genomes of 14 reference ST274-*P. aeruginosa* strains and control strains PAO1 and PA14 downloaded from the NCBI database; Table S3. Single-nucleotide polymorphisms (number of SNPs) in the core genome among the 11 studied ST274-*P. aeruginosa*, the 14 reference ST274-*P. aeruginosa* genomes, and the two control genomes; Table S4. Pangenome determined using Roary in all 25 ST274-*P. aeruginosa* strains and the control strains PAO1 and PA14; Table S5. Acquired genes in ST274-*P. aeruginosa* with Resfinder prediction; Table S6. Dataset and function of the 170 antimicrobial resistance genes analyzed to determine the mutational resistome of the ST274-*P. aeruginosa* strains; Table S7. Mutational resistome of the 25 studied ST274-*P. aeruginosa* strains with respect to PAO1 genome; Table S8. Detection of alterations in the mutome genes (involved in the mutator phenotype) of the 11 studied ST274-*P. aeruginosa* strains and the 14 reference ST274-*P. aeruginosa* genomes, with respect to PAO1 genome; Table S9. Dataset and functions of the 250 virulence genes analyzed to determine the virulome of the ST274-*P. aeruginosa* strains; Table S10. Amino acid changes in virulome of the 25 studied ST274-*P. aeruginosa* strains with respect to the PAO1 genome. References [80,81,82,83,84] are cited in the supplementary materials.
